# Renewing an old interest: Pituitary folliculostellate cells

**DOI:** 10.1111/jne.13053

**Published:** 2021-11-03

**Authors:** Paul R. Le Tissier, Patrice Mollard

**Affiliations:** ^1^ Centre for Discovery Brain Sciences University of Edinburgh Edinburgh UK; ^2^ Institute of Functional Genomics CNRS, INSERM University of Montpellier Montpellier France

**Keywords:** anterior pituitary, cell networks, folliculostellate cells, pulse generation

## Abstract

Anterior pituitary folliculostellate (FS) cells, first described almost 50 years ago, have a wide range of functions with respect to supporting and coordinating endocrine cell function, in particular through paracrine and gap junction‐mediated signalling. Our previous studies identified the morphological organisation of FS cells, which mediates coordinated calcium activity throughout the homotypic FS network and allows signalling across the whole pituitary gland. It is also clear that FS cells can modify endocrine output and feedback on pituitary axes over a range of timescales. Recently, several studies have defined FS cells as a source of anterior pituitary endocrine cell renewal, which has resulted in a renaming of FS cells as “Sox2+ve stem cells”. Here, we highlight the broader potential of the FS cell population in fine‐tuning and coordinating pituitary axes function. In addition, we identify a need for: the definition of the possible subtypes of FS cell and their relationship with the stem cell population; the potential role of FS cells in pulsatile hormone secretion and coordination of heterotypic cell networks; and the roles that FS cells may play in both early‐life programming of pituitary axes and in memory, or anticipation, of demand. Further studies of FS cells may demonstrate the fundamental importance of this cell type and its potential as a therapeutic target to correct pituitary gland dysfunction, one of which is stem cell therapy. Clearly, a thorough understanding of all of these interactions and relationships of FS and endocrine cells is required whatever therapeutic use is suggested by their various roles.

## Abstract 2

Pituitary folliculostellate cells have been studied for over 50 years, revealing their important but enigmatic function in anterior pituitary gland function. More recent research has focused on their potential as stem cells, although this should not override the importance of their other roles.

## 1 INTRODUCTION

Increasingly, neuroendocrine research focuses on its potential for an impact of novel findings on highly‐prevalent human health problems, in particular those where the underlying physiology and its dysfunction is poorly understood. Research on the pituitary, the master gland relaying hormonal information between the brain and many peripheral tissues, cannot escape this trend, as exemplified by the growing hope of regenerative medicine with the recent discovery of adult stem cells in the pituitary parenchyma. One exciting aspect of stem cell therapy is its potential to correct a number of pituitary disorders with aetiologies that are poorly understood. This focus on stem cells and their potential may explain how a subpopulation of pituitary cells, devoid of secretory granules, has changed from being described as folliculostellate (FS) cells (coined by Evelyne Vila‐Porcile in 1972^1^) to adult Sox2+ve stem cells. Recent studies have highlighted an important aspect of these stem cells, in that they are the source of secreted factors regulating not only their own function, but also those of other pituitary cells.^2^, ^3^ This aspect of the biology of pituitary stem cells resonates with roles described in previous studies of FS cells^4^ and has prompted us to reassess the biology of this enigmatic cell type and its importance for pituitary gland physiology. Because the stem cell potential of FS cells has been extensively reviewed recently,^5^, ^6^, ^7^ we focus here on other important aspects of FS cell biology in the regulation of anterior pituitary gland function.

## 2 FS CELLS AND THEIR CELL IDENTITY

**FIGURE 1:**
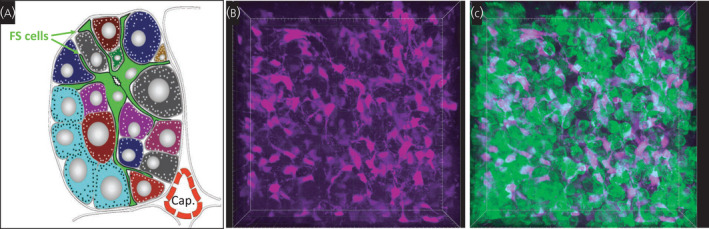
Pituitary folliculostellate (FS) cell organisation. A, Early studies of the morphological organisation of FS cells described them as star‐shaped cell, with cell bodies located among secretory cells and with processes extending between secretory cells and terminating at the perivascular space of capillaries (Cap.). Schema adapted from Perryman.^77^ B, C, Identification of FS cells in the lateral wing of a 2‐month‐old male growth hormone‐green flourescent protein (GH‐GFP) transgenic mouse, by labelling with dipeptide conjugated aminomethylcoumarin acetate (AMCA) (purple), demonstrates their organisation into a continuous network (B), which is intermingled with and makes multiple contacts with GFP‐labelled somatotrophs (C). 3D image stack (224 × 224 × 50 µm in the *x*‐, *y*‐ and *z*‐axis, respectively) acquired with two‐photon

FS cells were first described in the pioneering electron microscopy studies of the pituitary gland by Farquhar and Rinehart^8^ as small agranular cells with long, slender processes (Figure 1A). Although early work identified two types of cell,^9^, ^10^ these were unified as a single cell type by Vila‐Porcile, who also described their organisation into a network in the rat.^1^ Subsequently, they have been described in the anterior pituitaries in a range of mammalian and non‐mammalian species,^11^ as well as in anterior pituitaries with morphologically distinct organisation, such as teleosts.^12^ Classically, they have been studied in animals such as the rat, where their expression of S100, a family of Ca^2+^‐binding proteins transducing Ca^2+^ signals,^13^ enables identification based on gene expression as well as morphology.^14^ This expression of S100 has subsequently been exploited in the generation of green flourescent protein (GFP) transgenic rats, allowing the isolation and ready identification of these cells.^15^ FS cells can also be identified in *ex vivo* culture by their uptake of an alanine‐lysine dipeptide conjugated to aminomethylcoumarin acetate (AMCA) fluorophore, which is dependent on FS cell expression of a protein peptide symporter.^16^ Intriguingly, this uptake of dipeptide‐conjugated AMCA was shown to be a feature of posterior pituitary pituicyctes,^17^ which also express some of the key proteins related to FS cell function, suggesting that the distinct cell types of the two pituitary lobes may share common features and roles. More recently, specific pituitary expression of aldolase C in FS cells has been described in mice, which may allow increased genetic manipulation in this model species.^18^

Dispersed FS cells in culture readily self‐organise into aggregates of cells that resemble those of the intact pituitary and, in mixed cultures with other pituitary cell types, form clusters with hormone producing cells.^19^ A role for the chemoattractant molecule CXCL12 and its receptor CXCR4, both expressed by FS cells, has been described in this *in vitro* recapitulation of cell organisation.^20^ Homotypic FS cell interaction are then likely maintained by expression of adherence proteins such as E‐cadherin and a differential expression of other adherence molecules likely mediates specific FS cell–heterotypic cell morphological relationships.^21^ This suggests a role for FS cells in the organisation of the homotypic networks of all pituitary hormonal cell types studied to date, with important functional consequences.^22^ An important interaction of FS cells with the extracellular matrix (ECM) has also been described, with matrix metalloprotease 9 mediating cell organisation,^23^ integrin ß1 signalling regulating FS cell proliferation^24^ and FS cell production of tissue inhibitors of metalloproteinases in turn regulating the ECM.^25^

## 3 FS CELL NETWORK COMMUNICATION AND REGULATION OF ANTERIOR PITUITARY ENDOCRINE OUTPUT

### 3.1 FS cell network communication

The extensive FS organisation across the pituitary gland recognised by Vila‐Porcile,^1^ as well as the relationship with other pituitary hormonal cell types, led to a recognition that FS cells may mediate pituitary scale regulation and the coordination of endocrine output. We have investigated this using the uptake of dipeptide‐conjugated AMCA dye in *ex vivo* pituitary slices, allowing us to identify FS cells and, with the use of high‐resolution 3D microscopy that allows large‐scale reconstruction of cell organisation, we have shown that a network of connected FS cells wires the whole pituitary gland^26^ (Figure 1B). Furthermore, because the AMCA dye can be imaged in live cells, we were able to record calcium activity in FS cells, revealing spontaneous changes in cytosolic calcium, with a large proportion of cells firing monophasic calcium spikes. Communication across the gland is apparent as a wave of calcium activity that travels through the FS cell network from one wing of the pituitary to the other. Moreover, more localised cell–cell communication was evident within subsets of FS cells, suggesting specialised communication modes with endocrine cell neighbours.^27^ This calcium wave propagation is mediated by gap junctions between FS cells, which may also allow other small molecules (such as cAMP, inositol trisphosphate) to act as signals that can be transferred through the network.^26^ The role of this communication for FS cell function is currently unclear; however, pituitary cell organisation has been shown to have coordinating roles in a range of cell activities, including secretion and the regulation of gene expression.^28^

### 3.2 Communication with other pituitary cell types

An attractive hypothesis for a role of the FS cell network is in coordinating anterior pituitary hormone cell function, which is suggested by the morphological interdigitation of FS cells with network motifs of endocrine cells (Figure 1C). The importance of FS cells for normal pituitary function is exemplified by a recent study showing that FS cell dysregulation through the loss of Patched expression results in multiple alterations of anterior pituitary endocrine axes in adulthood.^29^ Gap junction‐mediated communication has been principally described as occurring within the homotypic FS cell network^30^; however, this does not preclude some communication by this mechanism with other cell types. Indeed, functional gap junctions between FS and pituitary hormonal cells have been reported.^31^, ^32^ Because gap junctions allow endocrine cell network propagation of calcium signals (similar to that shown for FS cells), only a low level of FS cell–endocrine cell communication may be required for a propagation through endocrine homotypic networks, resulting in the coordination of FS cell regulation on a much larger scale.

There has been much more extensive characterisation of paracrine communication between FS and endocrine cells. FS cells have been shown to produce a range of growth factors and cytokines, such as vascular endothelial growth factor (VEGF), basic fibroblast growth factor, annexin 1 (ANXA1) and interleukin‐6, as well as nitric oxide (NO),^4^ all of which have clear roles in the regulation of specific endocrine pituitary cell types. For example, in the embryo, NO has been shown to increase both growth hormone gene expression and the number of somatotrophs,^33^ whereas FS cell (intriguingly those near blood vessels) NO synthase expression has been shown to be increased by dopamine, potentially mediating some of the inhibitory actions of this small transmitter on prolactin release.^34^ To this list, we can now add the Wnt ligands because these have been shown to be secreted from Sox2‐positive stem cells.^2^ The alteration of many of these ligands in differing physiological states (see below) suggests an important role in the communication of FS with endocrine cells. It also raises the question of whether FS cells are either a single unique population (as proposed by Vila‐Porcile^1^) or a modular ensemble of FS cell subsets that adapt their range of paracrine communication in response to demands.

## 4 ROLE AS A RELAYER OF PERIPHERAL FEEDBACK SIGNALS?

Perhaps the most intriguing potential roles for FS cells, in terms of potential coordinators of endocrine function as well as stem cells, is their response to pituitary target organ feedback and mediators. This would place them as key modifiers of pituitary function that fine‐tune and adapt the various endocrine systems to ensure an optimal response to physiology, rather than as supportive cells as has been suggested previously.^35^ For example, in the prolactin axis, FS cells have been shown to mediate lactotroph proliferation in response to estradiol,^36^ an effect that is blocked by progesterone^37^; to have distinct morphological relationships with lactotrophs in seasonal breeding animals, with long FS cell processes wrapping around lactotrophs in the breeding season^38^; and to be increased in number in lactotroph‐tumour susceptible Fischer 344 rats compared to non‐susceptible strains.^36^ These effects are most likely primarily through paracrine interactions, especially because interleukin‐6 has a regulatory role on lactotrophs.^39^ However, the expression of enzymes and transporters in FS cells will alter the exposure of surrounding endocrine cells to feedback, such as type II iodothyronine deiodinase‐2 (Dio2) and monocarboxylate transporter 8 (MCT8), modifying triiodothyronine exposure,^40^, ^41^ and 11β‐hydroxysteroid dehydrogenase type 1, activating cortisone and increasing glucocorticoid feedback in the pituitary.^42^

Of the paracrine FS cell actions, perhaps the best characterised example is the modification of corticotroph function in response to glucocorticoid feedback through ANXA1. In the pituitary gland, ANXA1 is primarily produced by FS cells and is concentrated at points of FS–endocrine cell interaction.^43^, ^44^ Glucocorticoids increase FS cell ANXA1 expression resulting in translocation of the protein to the external surface of the plasma membrane.^45^ The externalised ANXA then binds to high affinity binding sites on corticotrophs,^46^ resulting in a reduction of adrenocorticotrophic hormone (ACTH) secretion in response to corticotrophin‐releasing hormone.^47^, ^48^ The regulation of ANXA1 activity may suggest one role for spatially localised calcium waves that we have described propagating through subsets of the FS cell network^26^, ^27^: the structure of ANXA1 has been shown to be calcium sensitive, with increased biological activity in the presence of Ca^2+^.^49^ The importance of ANXA1 in corticotroph regulation is also highlighted by a four‐fold increase in corticotroph cell number in male ANXA1‐knockout mice compared to wild‐type controls, although, interestingly, this effect is much less pronounced or even absent in females.^50^ Finally, FS cells may be programmed by early‐life exposure to glucocorticoids because ANXA‐1 expression is reduced in adult mice following prenatal dexamethasone exposure.^51^

## 5 PERSPECTIVES

If we combine the biology described above with their more recent description as pituitary stem cells, it is clear that FS cells have a central role in anterior pituitary biology and that these different facets of their activity should not be considered in isolation. As a non‐hormonal cell type that allows communication across the entire gland and has modifying functions on all endocrine cell types, FS cells are ideally placed to coordinate the various pituitary axes. In the remainder of this review, we highlight two aspects of FS cell biology that we consider important in elucidating their role in regulating pituitary hormone output: heterogeneity and pulse generation. We then speculate on the fundamental role that FS cells may have in fine‐tuning physiology ranging from reproduction to metabolism and stress. Each of these may be defined through dual recording of FS and endocrine cell activity in *ex vivo* slices, as well as targeting specific molecules and pathways and determining *in vivo* consequences. This will require cell‐type specific markers and targets, although the increasing availability of transcriptome data that includes FS cells suggests that these may be identified in the near future.

### 5.1 Heterogeneity

FS cells were originally identified as two distinct cell types, each agranular and characterised by long cytoplasmic projections, but with distinct morphological arrangements based on whether they surround cavities filled with colloidal milieu.^9^, ^10^ The morphological heterogeneity is further emphasised by differential protein expression and this has led many to question whether FS cells are a single cell type, both in developmental origin and in function.^4^ An excellent example of this and its importance is the pars tuberalis VEGF‐secreting FS cell population^52^: because VEGF is a target for treatment of cancer and other diseases,^53^ an understanding of the relationship of this cell population with other FS cells is required to recognise the potential side‐effects of VEGF therapy on pituitary function. Recent reports analysing single cells RNA sequencing (scRNAseq) of rodent anterior pituitary cells utilising unbiased clustering methods have variably described FS and Sox2+ve cells as single or multiple cell types.^54^, ^55^, ^56^, ^57^ It is important to note that differences in pituitary dissection, with the inclusion of the intermediate and posterior pituitary that contains cell types such as pituicytes and epithelial‐like cells expressing markers associated with FS cells,^58^ may affect the identification of potential subpopulations. Indeed, this recent description of the expression in pituicytes and epithelial‐like cells of markers used in previous analyses of FS cell function may require the reinterpretation of some older studies, in particular those using dispersed pituitary cells. However, there is a clear potential for FS cell subpopulations and this requires further analysis.

The heterogeneity of FS cells calls into question their common identity with stem cells: are they identical cell populations or is one a subtype of the other? Whether differential protein expression represents a distinct cell trajectory or simply transient differences in gene expression dependent on cell location and physiological status is currently unclear.^19^ If there is substantial overlap between FS and stem cell populations, then it is difficult to understand how they can have so many functions at the same time as maintaining a specialised stem cell role, especially because the stem cell population is depleted with age or following injury.^59^ In addition, if we are to understand the dynamic functional relationship between FS and other pituitary cell types, then elucidating the possible plasticity in FS cell roles becomes essential. For example, modelling and understanding thyrotroph function and how this relates to altered thyroid status requires a clear understanding of the role of the subset of FS cells found to express MCT8 and Dio2 in humans.^40^ The terminal differentiation of FS cells into distinct functional types also has important implications for their potential role in programming and memory (see below). Functional heterogeneity, whether permanent or transient, is increasingly becoming recognised in various pituitary cell types^60^; the importance in FS cell function may become apparent with further analysis of scRNAseq profiling of the transcriptome of the anterior pituitary, which may define subtypes of cells and possible specific ligand‐receptor co‐expression between certain FS cells and specific endocrine cells (or subsets).

### 5.2 Pulse generation

Perifusion studies of isolated pituitaries have shown that hormone output is spontaneously pulsatile in the absence of hypothalamic input.^61^, ^62^ Our descriptions of pituitary hormone networks^28^ have provided, in part, a mechanism where these spontaneous pulses could occur. However, it was the FS cell network that pioneered the description of the endocrine networks: the finding of spontaneous pulsatile activity in these cells, and in particular the identification of a proportion that apparently acts as a pacemaker,^26^ suggests that they may have a role. Indeed, the paracrine and gap junction‐mediated communication between FS and endocrine cells would provide a mechanism allowing the coordination of pulsatile release over different timescales. The interaction between endocrine and FS cells is, however, bidirectional, especially if peripheral feedback is considered, and the identification of which cell type is acting as a pulse generator is therefore complex. An example of this is FS cell modulation of gonadotrophin‐releasing hormone (GnRH) priming of luteinising hormone secretion from gonadotrophs^63^ which could not only be driven by long‐term paracrine or gap junction‐mediated signalling from FS cells, but also be a result of altered FS cell function in response to GnRH stimulation of gonadotrophs. This illustrates the concept that, for specific pituitary cell types, pulse generation may be primarily driven by hypothalamic factors (GnRH, growth hormone releasing hormone) with FS cells acting as a modulator. Indeed, modelling of the hypothalamic pituitary gonadal axis has suggested that altered FS cell function driven by oestradiol could have a role with respect to luteinising hormone surge driving ovulation.^64^ For the hypothalamic pituitary adrenal and thyroid axes, there is strong evidence that pituitary pulsatile release is possible with an invariant hypothalamic secretagogue^65^, ^66^ and FS cells may have a primary role in pulse generation. In the case of corticotroph output, modelling by Walker et al.^67^ has suggested that pulsatile ACTH can be driven by the delayed feedback of glucocorticoid, which would be consistent with the actions of glucocorticoid feedback on FS cell ANXA1. In thyrotrophs, the presence of thyroid‐stimulating hormone receptor on FS cells suggests an ultrashort feedback loop^68^ and the possible role of this in the dynamics of thyroid stimulating hormone output warrants consideration.

### 5.3 FS cell role in pituitary plasticity, memory and fine‐tuning function

Functional plasticity is an important feature of pituitary gland biology because the appropriate output of each pituitary hormone does not simply maintain homeostasis but is required to change dramatically in response to, as well as in anticipation of, physiological status. Modification of the number, size and morphological relationship of FS and endocrine cells in different physiological states,^38^, ^69^, ^70^ as well as with age,^71^, ^72^ has been described, suggesting an altered function, although it is possible that FS cells play a more fundamental role. Obviously, stem cell function allows FS cells to alter the number of endocrine cells and this is likely an important feature in the expansion of specific cell types in response to challenge, such as the expansion of the lactotroph population in pregnancy and lactation in humans^73^ or the thyrotroph population in response to hypothyroidism.^74^ An altered relationship of FS and endocrine cells, as occurs in seasonal breeding animals, will clearly change paracrine signalling relationships, although ECM‐remodelling and maintenance roles for FS cells may also have important implications for endocrine cell network organisation, signalling and hormone output. Altered gap junction‐mediated signalling does not require a morphological rearrangement or change in cell number and we have observed changes in gap junction distribution in the lactotrophs of lactating dams.^32^ Thus, each of the aspects of FS cell biology described here are likely to impact on the plasticity of gland function and further studies are likely to be inspired by scRNAseq experiments that characterise the altered gene expression of all cell types in response to physiological and pathological challenge.

The memory of previous physiological status is another key aspect of anterior pituitary gland biology. Excellent examples of this are the programming of the hypothalamic pituitary adrenal axis by prenatal and perinatal stress^75^ and memory in the prolactin axis of lactational demand.^32^ Previous studies of the response of FS cells to early‐life exposure to dexamethasone, showing alterations that persist until adulthood, suggest that fetal and early‐life programming of pituitary axes may be mediated by changes in FS cell function. This may also be possible in adult programming, or memory of demand, and the extent by which changes in FS‐endocrine cell relationships, including gap junction remodelling, may be permanently changed is unclear and warrants further study.

Finally, we would suggest that, in addition to roles in maintenance of homeostasis, plasticity and memory, FS cells are uniquely placed to fine‐tune functional output and coordinate the function of pituitary axes. Key to this may be the FS cell response and modification of target organ feedback through altered paracrine and gap junction‐mediated signalling, as well as modification of feedback signals such as glucocorticoids or thyroid hormone. In addition, the role of stem cells allows adjustment of the proportion of the different pituitary cell types, which may allow prioritisation of various axes with age; for example, increased growth hormone output at puberty which then declines with age. Two additional features of FS cell biology may be permissive to this fine‐tuning: the lack of expression of several receptors for hypothalamic secretagogues and the ability to signal throughout the pituitary independent of blood flow. Both of these may allow temporal coordination that otherwise would occur as a dorsal–ventral wave, as well as a degree of regulation that is independent of direct hypothalamic input. Again, pars tuberalis FS cell secretion of VEGF, mediating seasonal alterations of angiogenesis and lactotroph function,^52^, ^76^ may be another example of this, in addition to highlighting the diversity of FS cells in the pituitary.

## 6 CONCLUSIONS

The increasing interest in pituitary stem cells has highlighted the important roles that FS cells play in ensuring an appropriate output of hormone for regulation of a range of physiology. An understanding of each of these roles and how they are related is not only important for our fundamental understanding of physiology and disease, but also essential if these cells are to be targeted for therapeutic correction of pituitary deficiency or tumours. A key aspect of this is the question of whether FS and stem cells are a single population with a myriad of functions; if this is the case, then there may be a far‐reaching physiological impact of transplanting a large number of these cells into a pituitary for stem cell therapy.

Despite the lack of fundamental markers, as well as possible subtype and species differences, which has hindered studies of FS cells, there is an impressive body of evidence concerning their importance. However, this also highlights the need for further studies of what may turn out to be the most important aspect of all for pituitary cell types in terms of the coordinated regulation of fundamental processes. As interests in various cellular functions change with time, it is perhaps inevitable that cell types will change names, although it is nevertheless important to consider all of the functions of this cell type, regardless of whether they are called FS or Sox2+ve stem cells.

## CONFLICT OF INTERESTS

The author declares that they have no conflicts of interest.

## AUTHOR CONTRIBUTIONS

Paul Le Tissier: Conceptualisation; Writing – original draft. Patrice Mollard**:** Conceptualisation; Writing – original draft.

### PEER REVIEW

The peer review history for this article is available at https://publons.com/publon/10.1111/jne.13053.

## Data Availability

Not required.
